# The N-Terminal Extension of the *Mycobacterium avium* Rel Protein Is a Dual Regulator of the Bifunctional Enzyme and Represents a Novel Target

**DOI:** 10.3390/antibiotics14100964

**Published:** 2025-09-25

**Authors:** Tuck Choy Fong, Priya Ragunathan, Reema Alag, Carlos Silvester, Svarika Sonthalia, Vikneswaran Mathiyazakan, Vandana Grover, Gerhard Grüber

**Affiliations:** School of Biological Sciences, Nanyang Technological University, 60 Nanyang Drive, Singapore 637551, Singapore; tuckchoy001@e.ntu.edu.sg (T.C.F.); rpriya@ntu.edu.sg (P.R.); alagreema@gmail.com (R.A.); carlos.silvester@ums.edu.my (C.S.); svarika002@e.ntu.edu.sg (S.S.); vikneswaran.m@ntu.edu.sg (V.M.); vandana002@e.ntu.edu.sg (V.G.)

**Keywords:** mycobacteria, non-tuberculosis mycobacteria, *Mycobacterium avium*, stringent response, Rel, synthetase domain

## Abstract

**Background**: *Mycobacterium avium* (*Mav*) is a leading cause of pulmonary disease among non-tuberculous mycobacteria (NTMs) due to its extensive antibiotic resistance profile. The essential Rel protein is a bifunctional enzyme, which is sensitive to environmental stress and regulates cellular guanosine-3′,5′-bispyrophosphate ((p)ppGpp). Increased levels of the alarmone thereby initiate a survival response, contributing to bacterial persistence and virulence. **Objectives**: *Mav*Rel harbors an unusual extension at the N-terminal domain (NTD), which we aim to characterize its possible regulatory role in maintaining (p)ppGpp homeostasis. We also studied whether the TGS domain retains its regulation capacity in *Mav*Rel and the binding propensity of the ACT domain to valine. **Methods**: Molecular dissection of *Mav*Rel was performed to generate a series of truncates to quantify the synthetase and hydrolase activities. Binding experiments with tRNA and valine were carried out via tryptophan quenching assay and NMR, respectively. **Results**: Bi-catalytic regulation of *Mav*Rel was found to be predominantly governed by the residues 37–50 at the NTD extension in its free state. The TGS domain was shown to harbor the capacity to bind with deacylated tRNA and represses synthetase activity to a lower degree compared to the NTD extension. We also characterized the dimeric *Mav* ACT-domain and the interacting residues contributing to its affinity with valine to function as a nutrient sensor. **Conclusions**: The mapping of the unique NTD regulatory element of *Mav*Rel reveals its functional relevance to coordinate the catalytic states of synthetase and hydrolase, hence underscores the prospect to drive inhibitor development targeting this novel site against *Mav* infections.

## 1. Introduction

The *Mycobacterium avium* (*Mav*) complex (MAC) belongs to a class of non-tuberculous mycobacteria (NTMs), whereby this opportunistic pathogen comprises the substrains such as *M. avium*, *M. intracellulare*, and *M. chimaera* [1,2,3]. The prevalence of MAC infection is seeing an unprecedented increase globally, also accounting for the largest group of NTM isolates identified in patients [4]. The primary diagnosis of *Mav* infection is commonly linked to pulmonary disease [5] and is often concomitant with predisposing conditions, including bronchiectasis, pneumonia, and cystic fibrosis [6,7]. Owing to its ubiquitous nature [8], environmental risk factors further exacerbate human-to-pathogen association [7], including nosocomial infections [9], leading to a widespread global disease burden. The slow growing *Mav* [10] also delays clinical intervention as identification and susceptibility testing may take weeks to authenticate [11]. Moreover, intrinsic resistance, a primary limiting factor against antimicrobials, is largely attributed to its complex cell wall [12], biofilm formation [13], and the presence of efflux pumps [14]. Coupled with mutation-driven acquired resistance [15,16], treatment options against *Mav* are complexified, leading to prolonged combinatorial therapy regimens [17] with limited efficacy [18,19] and the possibility of recurrence [20]. Progress on drug development against NTMs is tepid or mostly repurposed from tuberculosis treatment [21], prompting the urgency to rejuvenate the pipeline of new chemical entities (NCEs) and address these unmet needs. Rational drug design requires identification of a suitable drug target that is crucial for bacterial survival. Therefore, the characterization of the target’s function and its physiological effect is paramount to uncovering potential key metabolic processes contributing to bacterial persistence.

**Figure 1:**
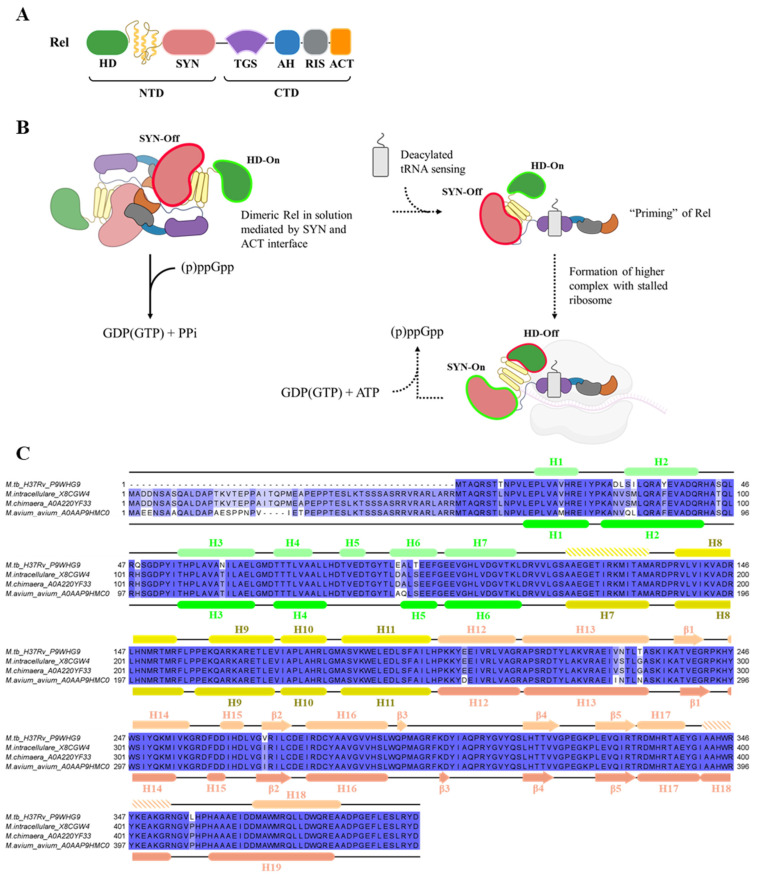
Features of mycobacterial Rel and its hypothesized regulatory mechanism. (**A**) Major domains of the Rel are broadly categorized as the catalytic active N-terminus domain (NTD) and regulatory C-terminus domain (CTD). Subdomains arrangement from N- to C-terminus are illustrated. HD (Hydrolase; green), alpha-helix linker (yellow), SYN (Synthetase; salmon), TGS (ThrRS, GTPase and SpoT; purple), AH (Alpha helix; blue), RIS (ribosome inter-subunit; gray), and ACT (Aspartokinase, Chorismate mutase, and TyrA; orange). (**B**) Proposed Rel reciprocal regulation of SYN and HD. Dimeric Rel (head-to-head arrangement) mediated by the SYN and ACT dimer-interface results in the TGS domain disrupting SYN active site while stabilizing HD conformation, inducing a SYN-Off/HD-On state. Under normal growth conditions (indicated by solid arrowheads), (p)ppGpp will be hydrolyzed into GDP(GTP) + PPi. In the state of nutrient deprivation pathway (indicated by hashed arrowheads), an increased level of deacylated tRNA is sensed by the TGS in Rel, which in turn promotes monomerization and then binds to tRNA to prime itself for ribosomal binding. The enzymatic state is postulated to maintain in the SYN-Off/HD-On conformation. Upon forming higher complex with the ribosome, the TGS and helical linker perturbs the accessibility of SYN and HD active sites, inversing the enzymatic states to SYN-On/HD-Off, thus promoting (p)ppGpp synthesis. (**C**) Amino acid sequence alignment of Rel from *Mtb* and the *M. avium* subspecies *M. intracellulare*, *M. chimaera*, and *M. avium* subsp. *avium* using Clustal Omega v2.3.0 [35]. Color intensity is proportional to the degree of consensus sequence. Secondary structures of *Mtb* (PDB ID: 5XNX; [36]) and *Mav* subsp. *avium*, derived from the structural model presented in this paper, are shown on the top and bottom of the alignment, respectively. HD domain (green), helical linker region (yellow), and SYN domain (salmon). Hashed bars in the secondary structures suggest probable helical elements which were not characterized in *Mtb*Rel.

The stringent response is a mode of bacterial adaptation against nutritional stress to invoke the regulation of (p)ppGpp (guanosine tetra- and penta-phosphate) alarmone levels in the cell [22]. Dynamic synthesis and hydrolysis of these signaling molecules are largely governed by the bifunctional Rel in mycobacteria upon sensing nutrient starvation, reflected by increased levels of deacylated tRNA [23]. Mycobacterial Rel enzyme comprises the N-terminal domain (NTD), where the catalytic synthetase (SYN) and hydrolase (HD) subdomains reside with the TGS- (ThrRS, GTPase and SpoT), AH- (Alpha helix), RIS- (ribosome inter-subunit), and ACT-subdomain (Aspartokinase, Chorismate mutase, and TyrA), forming the C-terminal domain (CTD) (Figure 1A) [24]. Thus far, only the *Mycobacterium smegmatis* (*Ms*) [11,12] and *Mycobacterium tuberculosis* (*Mtb*) [6,7,13,14,15] Rel had been described within the *mycobacterium* genus. Upon amino acid starvation, the dimeric Rel senses elevated levels of deacylated tRNA, undergoes monomerization, and complexes with the ribosome + tRNA to initiate (p)ppGpp synthesis via its SYN domain through a transferase reaction of the 5′-pyrophosphate from ATP to the 3′-OH of GDP or GTP [ATP + GDP(GTP) ⇌ AMP + (p)ppGpp] (Figure 1B) [25]. (p)ppGpp then initiates a cascade of regulatory pathways to control DNA replication [26], mRNA transcription [27], and translation [28] in a bid to conserve scarce energy resources and slow growth. In contrast, hydrolysis of the alarmone occurs when amino acid returns to basal level [(p)ppGpp ⇌ GDP(GTP) + PPi] [25] via its HD domain. Therefore, Rel is also inadvertently involved in the homeostasis of essential energy currencies such as ATP, accentuating its feasibility as a potential drug target.

Although the catalytic domains are confined within the NTD, allosteric regulation of the SYN and HD domains were characterized to be the central role of the CTD [29,30]. The TGS domain natively suppresses SYN and simultaneously upregulates HD activity via close contact remodeling of the SYN active site which, consequently, stabilizes the HD conformation [31]. During starvation stress, the TGS was elucidated via solution nuclear magnetic resonance (NMR) studies to show its capacity to bind with deacylated tRNA [23], which reverses the catalytic activities after binding to stalled ribosomes [31]. The trend of inverse activation/deactivation of the two active sites revealed the reciprocal SYN-On/HD-Off or SYN-Off/HD-On states (Figure 1B) [31,32]. In addition, the ACT domain acts as a secondary sensor by selectively binding to branched-chain amino acids (BCAAs), such as valine, triggering (p)ppGpp hydrolysis [33]. Another possible layer of indirect regulation entails the formation of a homodimer via the SYN/TGS [31] and ACT interface [34], which may restrict the accessibility of TGS to deacylated tRNA, whereas the ACT dimeric cleft enhances amino acid binding [34]. The importance of enzymatically active NTD and the regulatory CTD of Rel is therefore essential for mycobacterial persistence under growth stress. Precisely orchestrated switching of the synthetase and hydrolase activities allows for multi-step control of (p)ppGpp in the cell to manipulate growth rate at the replication, transcriptional, and translational levels. Here, we explore the role of the N-terminus 50 amino acid extension of the *Mycobacterium avium* as revealed via sequence alignment, which is absent in *Mtb* (Figure 1C), and its function as a specific regulator for the catalytic NTD. In addition, we decipher the differences between *Mav’s* TGS- and ACT-domains in enzyme regulation compared to other mycobacterial homologues and provide insights into tRNA and valine binding to their respective domains.

## 2. Results and Discussion

### 2.1. Construction of MavRel_1–444_ Prediction Model and Identification of Probable NTD Binding Motif

**Figure 2:**
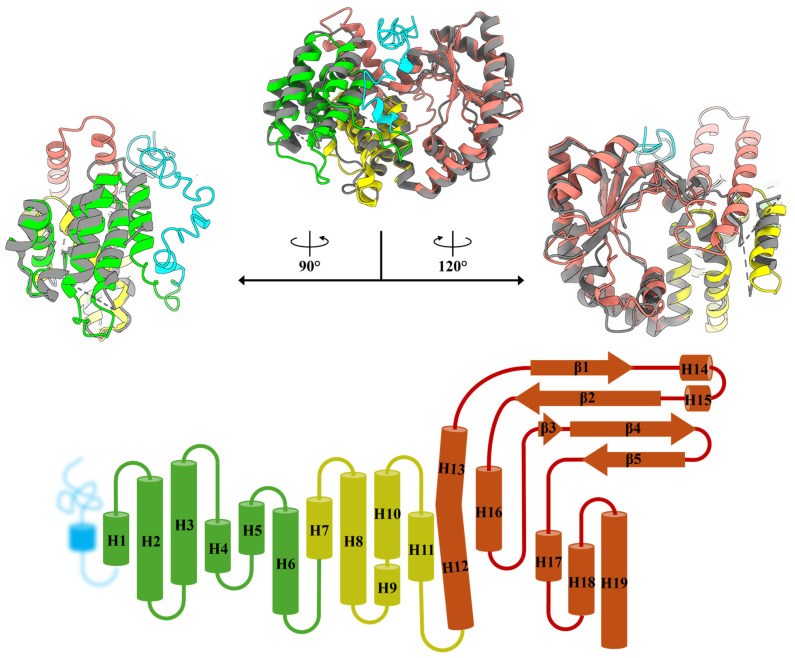
(**Top**) Model fitting of predicted *Mav*Rel_1–444_ (NTD extension, cyan; HD, green; alpha-helix linker, yellow; SYN, salmon) with the *Mtb*Rel_1–394_ (PDB: 5XNX [36]) in gray. Superimposition of the two structures demonstrated structural conformity on its overall architecture and domains arrangement where the r.m.s.d is calculated to be 1.185 Å. (**Bottom**) Schematic illustration of the *Mav*Rel NTD that is homologous to *Mtb*Rel NTD, except for the additional 50 amino acid extension in *Mav*Rel, which could not be accurately predicted, likely due to its intrinsically disordered nature.

The amino acid sequence alignment of Rel_1–444_ among the three subspecies of the MAC is almost identical (Figure 1C), where the hydrolase, helix bundle, and synthetase domain of *Mav* subsp. *avium* is homologous to the *Mtb* counterpart (95.7% similarity). This fact and the existing crystal structure of the nucleotide-free *Mtb*Rel_1–394_ (PDB ID: 5XNX) at 3.7 Å [36] enabled us to generate a homology model of the *Mav*Rel_1–444_ via I-TASSER [37] (Figure 2). The presented *Mav*Rel_1–444_ structure has an approximate full sequence coverage, excluding the 50 N-terminal residues of *Mav*Rel_1–444_, and demonstrates good correlation with the nucleotide-free *Mtb*Rel_1–394_ structure with a calculated root-mean-square deviation (r.m.s.d.) of 1.185 Å between C_α_ atoms (Figure 2). The previously uncharacterized NTD extension is presumed to dwell in the mid-section, between SYN and HD domains in this model.

**Figure 3:**
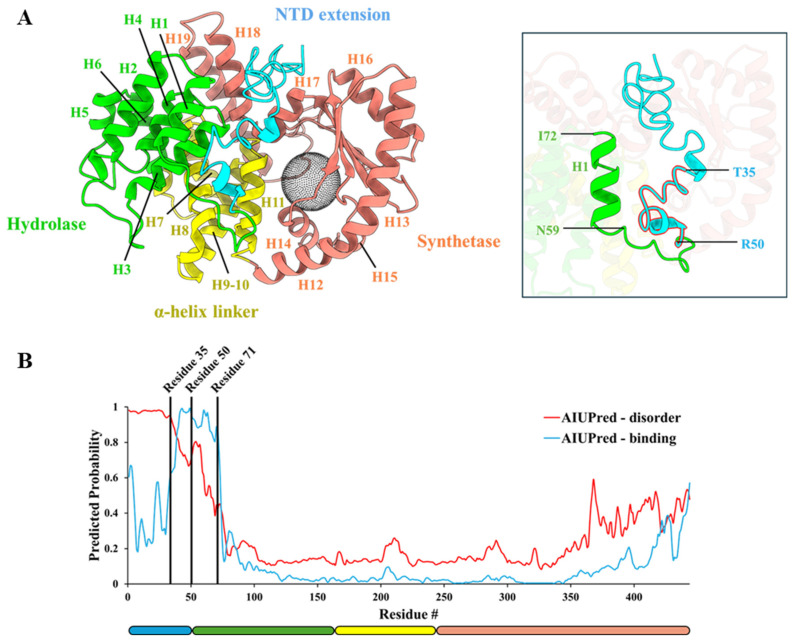
Probable binding motif in the amino acid extension of *Mav*Rel_1–444_. (**A**) Predicted structure of *Mav*Rel_1–444_ as derived by I-TASSER [37] depicts the possible spatial arrangement of the NTD extension on the cleft between the HD and SYN domains. Residues T35-R50 were observed to be in proximity to H1 of the HD domain. Binding site of the SYN domain is presented as a black sphere. (**B**) AIUPred [38,39] analysis of the *Mav*Rel_1–444_ suggests the first 50 amino acids are intrinsically disordered, but residues 35–50 may undergo structural changes to form a transient ordered element with a capacity to function as a binding motif. The domain regions are illustrated with respect to the residue number on the y-axis and colored as previously described.

The hydrolase (HD) domain of the *Mav*Rel_1–444_ model consists of helices α1-α6 and is connected via a helix bundle (α7–α11) with the synthetase (SYN) domain, composed of helices α12–α19 and β-sheets β1–β5 (Figure 2). The unique 50 amino acid N-terminal extension in *Mav*Rel, forms α-helical turns at amino acids S32–K34 and A45–R49, and a random coil structure between residues M1–E31 and T35–R44 in the *Mav*Rel_1–444_ model. The helical turn T35-R44 is in neighborhood to residues N59–I72 of the HD domain, while a major part of the N-terminal stretch is oriented to the cleft formed by helices α17 and α18 of *Mav*Rel_1–444′_s SYN domain (Figure 3A). To understand the innate structural characteristics of the largely polar 50 amino extension in the NTD, we probed for possible disorderedness with AIUPred [38,39] based on pairwise contact energy prediction [40,41]. Initial findings suggest that residues 1–34 are highly disordered, and the subsequent residues from 35 to 60 are comparatively more stable to potentially undergo a transient secondary structure formation such as an alpha-helix (Figure 3B). In conjunction with predictive binding analysis, residues 35–50 of the extension were estimated to be in favor of binding propensity for a disordered region. This prediction further extends to residue 71, which may suggest some form of interaction between the NTD extension and HD domain (Figure 3B). Considering the flexibility of the extra stretch with its α-helical turns and random coil feature, this structural model represents a possible arrangement of an ensemble of orientations within the dual enzyme domain and provides a starting point for the design of relevant mutational studies.

### 2.2. Expression and Isolation of MavRel NTD and Its Truncates

**Figure 4:**
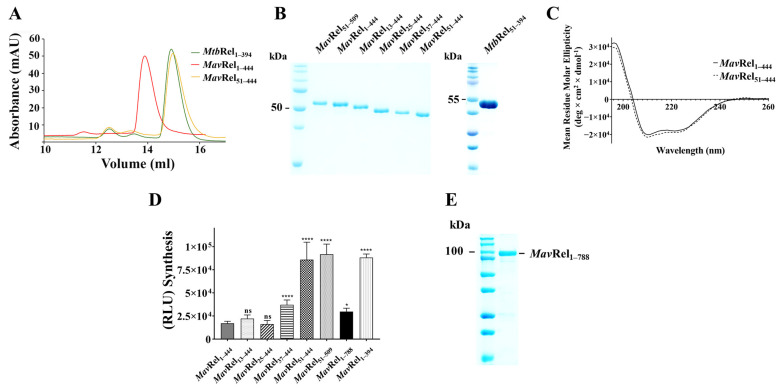
Purification and characterization of recombinant *Mav*Rel constructs. (**A**) SEC purification of *Mtb*Rel_1–394_, *Mav*Rel_1–444_, and *Mav*Rel_51–444_ presents similar elution profile in a Superdex 200 Increase 10/300 GL column, which corresponds to the dimeric *Mtb*Rel_1–394_ from the previous study [36]. (**B**) SDS-PAGE profiling of all *Mav*Rel NTD constructs displayed band sizes that are reflective to its truncation. (**C**) Far UV CD spectroscopy of *Mav*Rel_1–444_, and *Mav*Rel_51–444_. (**D**) Formation of AMP due to synthetase activity of *Mav*Rel_1–444_, *Mav*Rel_13–444_, *Mav*Rel_25–444_, *Mav*Re_37–444_, *Mav*Rel_51–444_, *Mav*Rel_51–509_, *Mtb*Rel_1–788_, and *Mtb*Rel_1–394._ The luminescence unit is directly proportional to the amount of AMP produced by the proteins in the presence of ATP and GTP. All data represented are the averages of two biological replicates with three technical replicates each. ****: *p* < 0.0001, *: *p* < 0.05, statistical analysis was carried out using ordinary one-way ANOVA (analysis of variance), ns: non-significant. (**E**) SDS-PAGE of WT *Mav*Rel showing a dominant single band approximating at 87 kDa.

To study whether this additional N-terminal stretch does affect catalysis of the *Mav*Rel_1–444_ SYN domain, recombinant *Mav*Rel_1–444_ and *Mav*Rel_51–444_, excluding the 50 amino acids stretch, were designed (Supplementary Figure S1) and expressed in *E. coli* C41(DE3) cells. *Mav*Rel_1–444_ and *Mav*Rel_51–444_ were produced in high amounts and purified using a Ni-NTA affinity followed by a size exclusion chromatography (SEC; Figure 4A). *Mav*Rel_1–444_ elutes at 14.1 mL and *Mav*Rel_51–444_ at 15.2 mL, similar to *Mtb*Rel_1–394_, which forms a dimer in solution as demonstrated by SEC and solution X-ray scattering [36], indicating that *Mav*Rel_1–444_ and *Mav*Rel_51–444_ are likely to be dimeric in solution and that the deletion of N-terminal 50 residues does not disrupt the dimer formation.

The SDS-PAGE (Figure 4B) of the eluted proteins verified the purity of *Mav*Rel_1–444_ and *Mav*Rel_51–444_. The circular dichroism (CD) spectra of *Mav*Rel_1–444_ and *Mav*Rel_51–444_, measured between 185 and 260 nm (Figure 4C), reflect proper folding of the secondary structures of both recombinant proteins with a major α-helical structure as well as the anti-parallel β-sheet content, making *Mav*Rel_1–444_ and *Mav*Rel_51–444_ suitable for activity studies.

### 2.3. The N-Terminal Extension of MavRel_1–444_ Affects Synthetase Activity

To quantify the activity of synthetase, a luminescence-based assay was employed to detect AMP production, which directly correlates with (p)ppGpp formation by *Mav*Rel_1–444_ and *Mav*Rel_51–444_. Figure 4D illustrates the relative luminescence, indicating *Mav*Rel_1–444_ synthetase activity at only 22% of *Mav*Rel_51–444_. Importantly, the synthetase activity of *Mav*Rel_51–444_ is comparable to the synthetase-active *Mtb*Rel_1–394_, which lacks the unique N-terminal residues 1–50 found in *Mav*Rel. The data underpin that the N-terminal 50 amino acids exert a major inhibitory effect on the SYN domain. To verify if this inhibitory effect is also present for WT (wild type) *Mav*Rel protein, the recombinant *Mav*Rel_1–788_ was expressed and purified (Figure 4E). As revealed in Figure 4D, *Mav*Rel_1–788_ shows a similarly low synthetase activity to *Mav*Rel_1–444_, which indicates that the N-terminal extra stretch inhibits (p)ppGpp synthesis in the entire *Mav*Rel, and that *Mav*Rel_1–444_ is a comparable model to study this phenomenon in more detail. To narrow the inhibitory motif of the N-terminal 50 residues, the truncates *Mav*Rel_13–444_, *Mav*Rel_25–444_, and *Mav*Rel_37–444_ were generated, purified, and characterized (Figure 4B,D). The synthetase activities of the deletion constructs showed that *Mav*Rel_13–444_ and *Mav*Rel_25–444_ maintained similar synthesis activity levels compared to *Mav*Rel_1–444_, while *Mav*Rel_37–444_ increased by 46%. In comparison to *Mav*Rel_51–444_, these data highlight the importance of residues 37–51 in regulating synthetase activity.

### 2.4. MavRel Is Critical for the Balancing of Currencies of Life

**Figure 5:**
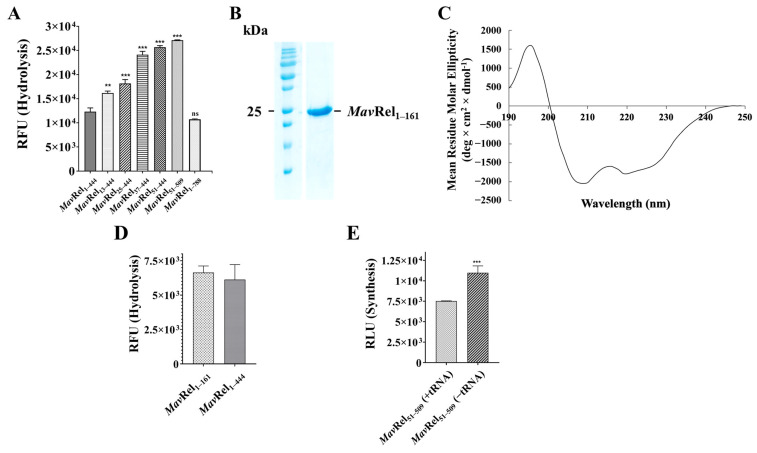
(**A**) pppGpp hydrolysis of *Mav*Rel_1–444_, *Mav*Rel_13–444_, *Mav*Rel_25–444_, *Mav*Re_37–444_, *Mav*Rel_51–444_, *Mav*Rel_51–509_, and *Mtb*Rel_1–788_. The emitted fluorescence intensity is proportional to the concentration of pyrophosphate. All data represented are the averages of two biological replicates with three technical replicates each. ***: *p* < 0.0007, **: *p* < 0.0046 statistical analysis was carried out using ordinary one-way ANOVA (analysis of variance), ns: non-significant. (**B**) SDS gel of the purified recombinant *Mav*Rel_1–161_. (**C**) CD spectrum of recombinant *Mav*Rel_1–161_, reflecting proper secondary structural content. (**D**) Comparison of hydrolytic activity of *Mav*Rel_1–444_ and *Mav*Rel_1–161_, demonstrating that the helix bundle and/or SYN domain have no major effect on pppGpp cleavage within the HD domain. (**E**) pppGpp synthesis of *Mav*Rel_51–509_ in the absence and presence of *E. coli* tRNA using a protein (monomer) to tRNA molar ratio of 1:2. ***: *p* < 0.0007.

The HD domain cleaves (p)ppGpp into GTP/GDP and PPi. Figure 5A shows that the concentration of pyrophosphate formed by *Mav*Rel_1–444_ and the entire enzyme *Mav*Rel_1–788_ are similar, indicating that both constructs are hydrolytically active and that *Mav*Rel_1–788′_s C-terminus has no major regulative influence on the HD domain. To rule out any effect of the neighboring helix bundle and/or SYN domain on (p)ppGpp cleavage by the HD domain, the *Mav* HD domain, including the 50 amino acid N-terminal stretch (*Mav*Rel_1–161_), was generated (Supplementary Figure S1) and purified (Figure 5B). As revealed in Figure 5C, *Mav*Rel_1–161_ shows proper secondary structural content as indicated by the CD spectra. Importantly the hydrolytic activity of *Mav*Rel_1–161_ was comparable to *Mav*Rel_1–444_ (Figure 5D). This result excludes any cooperative regulation via the helix bundle and/or SYN domain.

While we observed that deleting the first 37 residues (*Mav*Rel_13–444_, *Mav*Rel25–_444_, and *Mav*Rel_37–444_) of the N-terminal stretch did not significantly impact (p)ppGpp synthesis (Figure 4D), we addressed whether this flexible part of the N-terminal stretch may instead affect the HD domain. As illustrated in Figure 5A, the N-terminal deletion variants *Mav*Rel_13–444_, *Mav*Rel_25–444_, and *Mav*Rel_37–444_ showed an increase in PPi generated up to 95%, while no further increment was observed in the *Mav*Rel_51–444_ variant. The results further imply that the specific N-terminal extension plays a dual regulatory role, whereby its flexibility and relative orientation could enable the control of both catalytic domains to keep the balance of synthetase and hydrolase activities, enhancing energy utilization efficiency. Regulation of a bifunctional enzyme by an unstructured extension has also been described for *Mtb* [42,43], *M. smegmatis* [44,45], and the *Mycobacterium abscessus* F_1_F_O_-ATP synthetase subunit α [46]. Cryo-EM studies visualized that the unstructured mycobacterial specific C-terminus is flexible and shifts into a β-strand when binding to the rotary motor element of the enzyme [43,45,46]. Combining in silico efforts, the novel anti-TB inhibitor AlMF1 was identified [47]. Considering the dimeric form of *Mav*Rel_1–444_ in solution and the proposed mycobacterial head-to-head arrangement (Figure 1B), one could hypothesize that each of the two N-terminal stretches of a monomer could act like a switch and move between its own HD and SYN domain or the bifunctional domain of the second monomer to alter activity.

### 2.5. TGS Domain Does Not Affect Mav’s Bifunctional NTD Domain

The *Mtb*Rel [36] and *B. subtilus* Rel [31] TGS domain interact via a hydrophobic patch with the SYN domain and have been proposed to regulate the synthetase state [31]. Here, we designed, produced, and purified *Mav*Rel_51–509_, inclusive of the TGS domain (Figure 4B and Figure S1), to study whether the *Mav* TGS domain may alter synthesis or hydrolysis of (p)ppGpp in the absence of the extra N-terminal stretch. The comparison in Figure 4D demonstrates that the TGS domain of *Mav*Rel_51–509_ does not reduce the high synthetase activity of *Mav*Rel_51–444_. Furthermore, the hydrolytic activity of (p)ppGpp to GTP and PPi of *Mav*Rel_51–444_ is also not altered in *Mav*Rel_51–509_ (Figure 5A). In the next step, we asked whether deacylated *E. coli* tRNA, shown to bind to the *Mtb* TGS [23] and inhibit the SYN domain of *Mav*Rel_51–509_. As shown in Figure 5E, the presence or absence of tRNA at a molar ratio of 1 (monomeric Rel):2 (RNA) reduced synthetase activity by about 36%. These data underscore that the *Mav*Rel extra N-terminal stretch is the main intrinsic regulatory element of *Mav’s* SYN and HD domain in the absence of deacylated tRNA. The binding of tRNA to the TGS domain contributes in part to an allosteric regulation in the SYN domain to initiate a SYN-OFF state. Whether tRNA binding may further trigger the bifunctional domains in a *Mav*Rel-ribosome complex, however, cannot be ruled out.

### 2.6. TGS Domain Binds to the tRNA

**Figure 6:**
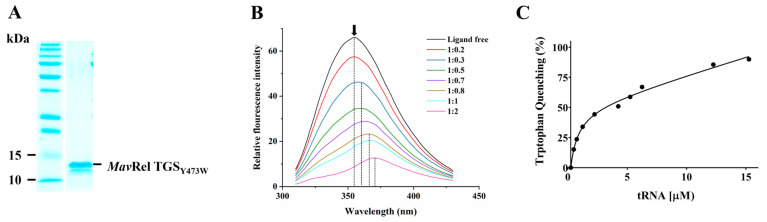
(**A**) 12% SDS-PAGE of purified *Mav*Rel TGS_Y473W_ mutant for tryptophan quenching assay. (**B**) Intrinsic tryptophan fluorescence spectra of *Mav*Rel TGS_Y473W_ in the absence and presence of increasing concentrations of tRNA showing tryptophan fluorescence quenching. Red shifts in the emission maxima (dotted lines) suggest a change in chemical environment or conformation around Y473W residue. (**C**) Fluorescence titration of *Mav*Rel TGS_Y473W_ at increasing concentrations of tRNA (0–15 μM) to determine the tRNA binding constant.

To confirm tRNA binding and determine its affinity to the TGS domain, a recombinant protein with Y473 substitution to a tryptophan (*Mav*Rel TGS_Y473W_) was designed, expressed, and purified (Figure 6A and Figure S1). *Mav*Rel TGS_Y473W_ was proposed, considering that the *Mtb* Y423 counterpart has been shown to be involved in tRNA binding to *Mtb*Rel [23] and to utilize the intrinsic tryptophan fluorescence in our fluorescence quenching study.

Upon excitation at 295 nm, the tryptophan residue emits fluorescence with a peak emission (λ_max_) at 355 nm (Figure 6B). Ligand-induced structural changes near the tryptophan residue could alter both the fluorescence intensity and the emission wavelength, reflecting changes in the local environment [48]. The emission spectrum of *Mav*Rel TGS_Y473W_ confirmed a λ_max_ at 355 nm upon excitation at 295 nm (Figure 6B). Titration with increasing concentrations of tRNA resulted in a progressive quenching of fluorescence at this wavelength. Notably, when the monomeric *Mav*Rel TGS_Y473W_:tRNA molar ratio exceeded 1:0.5, a red shift in the emission maximum was observed, suggesting increased hydrophilicity or conformational changes around the tryptophan residue upon binding. Quantitative analysis of the binding curve yielded a dissociation constant (*K_d_*) of 0.65 ± 0.2 μM, indicating a moderate to high affinity of *Mav*Rel TGS for tRNA (Figure 6C). The *K_d_* was determined by nonlinear regression using a one-site binding model. The best-fit *K_d_* was 0.65 µM, with a standard error of 0.19 µM and a 95% confidence interval of 0.18–1.12 µM. The quality of fit was high (R^2^ = 0.994; standard deviation of residuals = 2.76; degree of freedom = 6). Additional fit parameters included B_max_ (50.2 ± 4.7) and background (0.17 ± 0.08). Considering the nano-molar binding and *K_d_* determined, the drop of pppGpp synthesis (36%) in the presence of *E. coli* tRNA (1:2 molar ratio) is moderately suppressed.

To confirm tRNA binding also in the full-length enzyme, we repeated the same experimental setup for the *Mav*Rel Y473W mutant (Supplementary Figure S2A). The fluorescence spectra presented a similar quenching profile to *Mav*Rel TGS_Y473W_, with the *K_d_* determined at 1.67 ± 0.2 μM (Supplementary Figure S2B,C). We then conclude that the dimeric association of *Mav*Rel has negligible effect on tRNA binding to the TGS domain.

### 2.7. MavRel ACT Interacts with Valine

**Figure 7:**
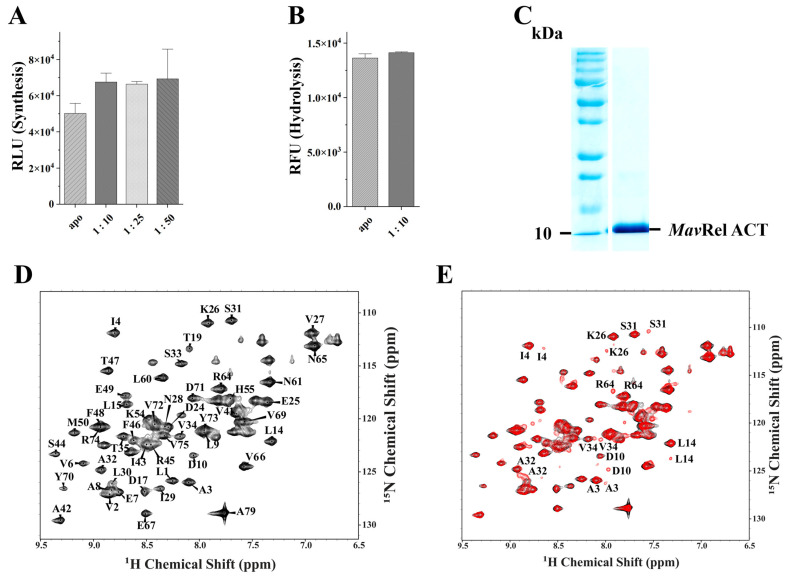
(**A**) Synthesis of pppGpp of WT *Mav*Rel_1–788_ was activated via the addition of valine at 1:10 molar ratio by approximately 45%, while further increment of valine does not appear to upregulate the activity. (**B**) pppGpp hydrolysis was not perturbed by the presence of valine when compared to WT *Mav*Rel in apo-form. (**C**) SDS gel of the purified recombinant *Mav*Rel ACT domain. (**D**) ^1^H–^15^N HSQC assigned spectrum of *Mav*Rel ACT, with well-dispersed peaks indicating proper folding of the protein. (**E**) Interaction of *Mav*Rel ACT with valine amino acid. Overlay of ^1^H–^15^N HSQC spectra of apo *Mav*Rel ACT (black) and *Mav*Rel ACT with Valine amino acid (red) at protein to amino acid ratio of 1:10. Protein residues showing chemical shift perturbations as well as appearance of new peaks in non-overlapping regions have been labeled.

Amino acid binding to the ACT domain triggers the bacterial Rel-ribosome interaction by regulating the bi-catalytic domains in the presence and absence of the amino acid [33]. In the case of *Mav*Rel (*Mav*Rel_1–788_), the C-terminus, including the TGS domain, does not affect (p)ppGpp synthesis (Figure 4D) or hydrolysis (Figure 5A) in the absence of valine. However, (p)ppGpp synthesis by *Mav*Rel_1–788_ increases in the presence of valine by about 35% at approximately 1:10 molar ratio of enzyme (monomeric):valine; thereafter, it was not perturbed by any further increment of amino acids (Figure 7A). In contrast, the addition of valine does not change the hydrolase activity of the enzyme (Figure 7B). The data demonstrate that *Mav*Rel becomes moderately stimulated by valine and may contribute to amino acid signaling under starvation conditions.

Valine binding to the dimeric *Mtb* ACT has previously been mapped using solution NMR spectroscopy [34]. Here, we generated and purified the *Mav*Rel ACT domain and its ^15^N labeled form, including residues L1-A79 (equivalent to amino acids L710-A788 in full-length *Mav*Rel; Figure 7C), using an affinity and size-exclusion chromatography (see Section 4). The 2D ^1^H–^15^N HSQC spectrum of ^15^N labeled *Mav*Rel ACT showed a well dispersed high-quality spectrum of 60 peaks, an indicator of a properly folded protein (Figure 7D). Based on HNCA, HNCACB, CBCA(CO)NH, and HN(CO)CA spectra, as well as selective labeling experiments for the amino acids, valine, alanine, leucine, isoleucine, and threonine, 55 out of 60 visible peaks in HSQC were assigned, leading to 91.6% assignment completion.

**Figure 8:**
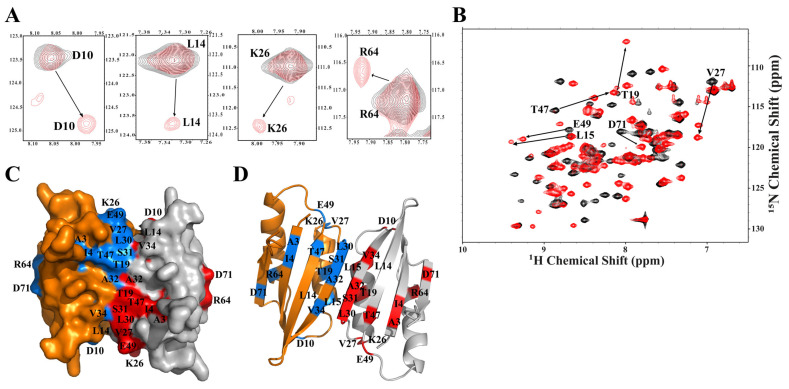
(**A**) Chemical shift perturbations and appearance of new peaks at protein (dimer) to valine molar ratio of 1:10. *Mav*Rel ACT residues such as D10, L14 and K26, involved in valine binding, show CSPs and emerging of new peaks. R64 showing CSPs due to indirect effect of binding. (**B**) Overlay of HSQC spectra of apo *Mav*Rel ACT (black) and valine bound *Mav*Rel ACT (red) at protein (dimer) to valine molar ratio of 1:50. Mapping of *Mav*Rel ACT residues showing significant change in CSPs involves L15, T19, V27, E49, T47, and D71 residues. (**C**) Surface representation of *Mav*Rel ACT dimer. Each dimer is shown in different colors (orange and gray). Residues involved in direct or indirect binding to valine, are shown in blue and red for each unit and present at dimeric interface of the protein. (**D**) Valine binding cleft consists of α1 helix, loop and β2 strand, and some other extended region of the protein. The structure is visualized using PyMol v3.1 [49].

To study *Mav*Rel ACT-valine interaction, ^1^H–^15^N HSQC titration experiments were performed with protein (dimer) to amino acid molar ratios of 1:10 and 1:50. The appearance of new small peaks, while the apo form peaks are still present (molar ratio of 1:10), indicates *Mav*Rel ACT:valine binding as well as a change in conformation (Figure 7E). Many residues, including A3, I4, D10, L14, K26, L30, S31, A32, V34, and R64, revealed a change in chemical shifts and appearance of new weak peaks. Our previous study with *Mtb*Rel ACT showed that D10-L15 and K26-L30 form a binding cleft for valine amino acid [34], and other *Mtb*Rel ACT amino acids, including A32-V34, S44, M50, R64, V69, and D71, might be indirectly affected by valine binding, as reflected by chemical shift change and new peak appearances [34]. Here, we demonstrate that valine binding to *Mav*Rel ACT occurs in a similar binding cleft formed by residues D10, L14, and K26, like in *Mtb*Rel ACT, going along with changes in chemical shifts and formation of new peaks (Figure 8A). In addition, the chemical shift in R64 (Figure 8A) might indicate an indirect effect caused by conformational changes after valine binding.

Furthermore, to saturate the protein with valine, we performed an NMR titration experiment at a 1:50 protein to valine ratio, where significant chemical shift perturbations (CSPs) were observed for many residues. To identify the residues showing significant changes in CSPs, we traced the residues from the previous studies of binding between *Mtb*Rel ACT and valine [34]. We found that residues L15, T19, T47, E49, and D71 show big changes in CSPs (Figure 8B). Significant changes in CSPs of *Mav*Rel ACT upon valine binding indicate direct interaction of these residues in valine binding and/or reflect conformational changes in this area due to valine binding.

Analysis of residues involved in interaction with valine on the protein surface of the Swiss-Model-generated dimeric model of *Mav*Rel ACT, with an RMSD of 0.244 Å when superimposed on the *Mtb*Rel ACT [34], highlighted that most of the residues are present in and around the dimeric interface of the antiparallel *Mav*Rel ACT dimer (Figure 7C). NMR titration data revealed that the helix α1 residues L14, L15, T19, loop residues K26 and V27, and β2 residues L30, S31 and A32 form the binding cleft and are also part of the *Mav*Rel ACT dimer interface (Figure 8C,D), consistent with the previous studies of *Mt*Rel ACT:valine [34]. Furthermore, the valine binding to the *Mav*Rel ACT cleft sequentially affects the residues V34, T47, E49, A3, I4, R64, and D71 (Figure 8C,D), indicating an extended conformational transition in *Mav*Rel ACT. These NMR results revealed the role of dimeric interface in valine binding.

## 3. Conclusions

Synthesis and hydrolysis of (p)ppGpp changes bacterial physiology, including down-regulation of rRNA synthesis; affecting poly-phosphate metabolism, gene regulation, and purine metabolism; up-regulation of protein degradation and amino acid biosynthesis; as well as shifting the bacteria to a non-replicating state [50]. Furthermore, it also alters ATP, GDP, and GTP homeostasis. Therefore, regulation of (p)ppGpp formation is of greatest importance for the *Mav* complex. The identification and mapping of the new N-terminal regulatory element of *Mav*Rel describe a novel mechanism of regulation of the bi-catalytic enzyme, which may enable proper management of the substrates and currencies of life, ATP and GTP [51], with the latter being essential for ribosomal function and therefore protein synthesis. The data may contribute to the design of molecules disrupting the interactions of the N-terminal stretch with the HD and SYN domains to interrupt the process of stress signaling and nucleotide homeostasis.

The pursuit of drug development on intrinsically disordered proteins (IDPs) is unconventional [52,53,54,55,56] as described above for the unstructured C-terminus of the mycobacterial F-ATP synthase subunit α [46]. IDPs are mostly involved in signal transduction and regulatory mechanisms [57], where it often undergoes a disorder-to-order transition [57,58]. This transition is a result of a large entropy cost [53], leading to a specific but weak affinity interaction [59,60], allowing for a disordered motif to become intrinsic to function [54]. Their free energy profile is thus vital for delicate regulatory processes such as a molecular switch that requires binding interaction to be reversible and transient [56,61]. Consequently, the presence of a specific inhibitor can readily displace the IDP to disrupt protein function [59]. One classical example is the p53-MDM2 mimetic, Nutlins [62,63], which competitively dock on the intrinsically disordered binding interface [52]. Despite the major challenge of IDPs being the lack of stable conformation for structural analysis [64], their structural plasticity confers functional advantages with its large binding surface and contact points [55]. Advances in computational modeling have now allowed molecular dynamics simulations to adopt an ensemble docking approach with improved sampling techniques [56] to predict IDPs’ motility and binding capacities [52]. Supporting methodologies such as small-angle X-ray scattering (SAXS), NMR, Förster resonance energy transfer (FRET), or different conformers resolved by cryo-electron microscopy can further elucidate ligand-IDP interactions [56].

Additionally, the experimental evidence presented showed that *Mav’s* TGS domain does not regulate the bi-catalytic *Mav*Rel as described for the *Mtb* enzyme [65], emphasizing the variance in Rel’s regulatory mechanism, in comparison to the *Mtb* and *B. subtilus*. However, *Mav’s* TGS domain bound to deacylated tRNA is proposed to allosterically induce in part a SYN-OFF state. The study shows for the first time valine binding of the *Mav* ACT domain and allowed mapping of amino acids involved in valine-to-*Mav* ACT binding. While amino binding to the ACT domain may in part trigger (p)ppGpp formation and *Mav’s* Rel-ribosome interaction, the ACT domain does not alter (p)ppGpp synthesis or hydrolysis in the absence of the amino acid as described for other bacterial counterparts, underscoring the major regulatory role of *Mav’s* Rel N-terminal stretch and its attractions as a novel target for new hit identification.

## 4. Materials and Methods

### 4.1. Structure Prediction and Modeling

Prediction and homology modeling of *Mav*Rel_1–444_: The structural model of the N-terminal domain (NTD) of *Mav*Rel, called *Mav*Rel_1–444_, was generated using the gene sequence of *relA* from *M. avium* subspecies *avium*. The 3D structure modeling was performed using I-TASSER [37], where the structure with the best C-score of 0.95 was selected for prediction model study.

Structural modeling of *Mav*Rel ACT domain: Dimeric *Mtb*Rel ACT structure (PDB ID: 6LXG [source]) was input as the base template for *Mav*Rel ACT homology modeling using SWISS-MODEL [29]. The predicted model generated was evaluated for Global Model Quality Estimate (GQME) with a score of 0.8, presenting reliable quality estimate of the summed per-residue.

Predictive analysis of intrinsically disordered regions: The analysis was performed on AIUPred [38,39] web server to identify disorderedness based on contact energy predictions. Binding propensity was calculated according to ANCHOR2 algorithm [66].

### 4.2. Generation of MavRel WT, NTD Constructs, Y473W Substitutions, and TGS/ACT-Domain Isolates

The *relA* gene encoding the *Mav*Rel protein of *Mycobacterium avium* subsp. *avium* (strain ATCC: 25291; Taxonomy ID: 44454) was cloned into pET29b and synthesized by Twist Bioscience (South San Francisco, CA, USA). A His_6_-tag was incorporated at the 5′—end of *relA*’s NTD in the design of the construct to enable downstream purification of the recombinant protein by Ni-NTA affinity chromatography. To generate pET9d-*Mav*Rel_51–788_ construct without the N-terminal 50 amino acid stretch, the coding region for the residues 51–788 of *Mav*Rel was amplified from genomic DNA by polymerase chain reaction (PCR) using forward primer 5′-ATGGGGATGACCGCCCAGCGCA-3′ and the reverse primer 5′-CGGATCCTCAGGCGGCGGAGGTCAC-3′. The pET9d backbone vector was isolated from pET9d-*Mt*Rel plasmid using the forward primer 5′-CGCCTGAGGATCCGGCTGCTAACAAAGCC-3′ and the reverse primer 5′-GGCGGTCATCCCCATGGGGTGATGGTGAT-3′. Subsequently, these fragments were ligated using NEBuilder^®^ HiFi DNA Assembly according to manufacturer’s protocol (New England Biolabs, Ipswich, MA, USA).

The genes encoding the *Mav* Rel truncates and substitutions (Supplementary Figure S1) were amplified with its respective templates and the primers listed in Supplementary Table S1. Following PCR amplification, the DNA template was digested from the PCR products using Dpn1 treatment. Subsequently, the modified DNA samples were introduced into *Escherichia coli* (*E. coli*) Top10 cells for plasmid amplification. Verification of plasmids containing mutations were then validated via DNA sequencing (Bio Basic Asia Pacific, Singapore).

### 4.3. Expression and Purification of MavRel WT and Its Mutants

Isolation of *Mav*Rel WT, NTD truncates and *Mav*Rel Y473W: Plasmids containing the target gene were introduced into electrocompetent *E. coli* C41(DE3) cells and subsequently cultured on Luria–Bertani (LB) agar plates enriched with 30 µg/mL of kanamycin. Single colonies were selected and inoculated into LB liquid media containing 30 µg/mL of kanamycin. Cells were cultured at 37 °C and 180 rpm until the optical density at 600 nm (OD_600_) reached 0.6, and the protein expression was induced by the addition of 1 mM isopropyl-ß-D-1-thiogalactopyranoside (IPTG) at 18 °C overnight. Cells were harvested by centrifugation at 4 °C, 6500× *g* for 10 min.

Cells were resuspended in buffer A (50 mM Tris/HCl, pH 8.5, 750 mM NaCl, 10% glycerol), 2 mM Pefabloc^SC^, 1 mM dithiothreitol (DTT), and 2 mM phenylmethylsulfonyl fluoride (PMSF), and lysed on ice using sonication. The cell lysate was subsequently clarified via centrifugation at 13,000× *g* for 30 min, filtered (0.45 µm; Millipore, Darmstadt, Germany) and incubated with Ni-NTA Agarose beads (QIAGEN, Hilden, Germany) for 1 h at 4 °C. The respective His_6_-tagged proteins were eluted by incrementally increasing imidazole concentrations from 0 to 450 mM in buffer A. The eluted fractions containing the desired protein were combined prior to injection onto a size-exclusion chromatography (SEC; Superdex™ 200 Increase 10/300 GL column; Cytiva, Marlborough, MA, USA) in buffer B (50 mM Tris/HCl, pH 8.5, 350 mM NaCl, 5% glycerol, 1 mM DTT). The respective recombinant proteins were then concentrated using a 30 kDa cut-off centrifugal unit (Millipore, Burlington, MA, USA) at a centrifugal speed of 4000× *g*. Recombinant *Mtb*Rel_1–394_ was purified according to Singal et al. [36]. The protein concentration was measured at 280 nm utilizing a NanoDrop™ 2000 spectrophotometer (Thermo Fisher Scientific, Waltham, MA, USA). The extinction coefficient and molecular weight of the protein were determined using the online program PROTPARAM [19]. Protein purity was evaluated using SDS-PAGE [67].

Purification of TGS/ACT domain and *Mav*Rel TGS_Y473W_: Purification of the two domains was performed as follows: Cells were lysed in buffer A containing 50 mM Tris/HCl, pH 8.5, 750 mM NaCl, 10% glycerol, 2 mM Pefabloc^SC^, 1 mM DTT, and 2 mM PMSF. Firstly, the cell lysate was centrifuged at 13,000× *g* to remove cell debris. Thereafter, the supernatant was passed through a 0.45 µm filter and incubated with Ni-NTA Agarose beads for 1 h at 4 °C. Ni-NTA affinity elution (with imidazole gradient 0–450 mM) was performed in buffer B (50 mM Tris/HCl, pH 8.5, 350 mM NaCl, 5% glycerol, and 1 mM DTT), and SEC (Superdex™ 75 10/300 GL column; Cytiva, Marlborough, MA, USA) was performed in buffer B containing 50 mM Tris/HCl, pH 8.5, 350 mM NaCl, 5% glycerol, and 1 mM DTT. The eluted recombinant proteins were then pooled and concentrated with 3 kDa centricon (Millipore, USA). ^15^N, ^13^C-^15^N, and selectively labeled (valine, alanine, leucine, isoleucine, and threonine) *Mav*Rel ACT domain were purified as described before [34]. For NMR titration experiment, SEC was performed in buffer C containing 50 mM Tris/HCl, pH 8.5, 100 mM NaCl, and 5% glycerol. All NMR samples included 10% D_2_O along with other buffer B components.

### 4.4. CD Spectroscopy

Steady-state circular dichroism (CD) spectra were measured in the far-UV-light range (180 to 260 nm) using a Chirascan spectrometer (Applied Photophysics, Surrey, UK). Spectra were collected in a 60-μL quartz cell (Hellma, Müllheim, Germany) with a path length of 0.1 mm at 20 °C and a step resolution of 1 nm. The readings were averages of 2 s at each wavelength, and the recorded millidegree values were averages of 3 determinations for each sample. CD spectroscopy of *Mav*Rel_1–444_, *Mav*Rel_51–444_, and *Mav*Rel_1–161_ (1.0 mg/mL) was performed in a buffer consisting of 50 mM Tris at pH 8.5, 350 mM NaCl, 5% glycerol, and 1 mM DTT. The spectrum for the buffer was subtracted from the spectrum of the protein. CD values were converted to mean residue molar ellipticity (θ) in units of degrees square centimeters per decimole per amino acid using Chirascan software (version 1.2; Applied Photophysics). The CD spectra were analyzed as described previously [34].

### 4.5. Synthetase Activity Assay

The catalytic activity of all recombinant proteins was confirmed using the endpoint synthetase assay AMP-Glo™ from Promega (Madison, WI, USA). The reaction buffer was set up by resuspending ATP (100 µM), GTP (100 µM), MgCl_2_ (200 µM), bovine serum albumin (BSA; 0.1 mg/mL), and DTT (1 mM) in 50 mM Tris at pH 8.5, 350 mM NaCl, and 5% glycerol. To generate the standard curve, an AMP sample gradient ranging from 20 to 0.625 µM was prepared by serially diluting a 10 mM AMP stock from the assay kit into the reaction buffer. All recombinant constructs were tested at concentration of 2 µM in the reaction buffer. The different proteins were incubated with the reaction buffer for 1 h at room temperature to initiate the formation of (p)ppGpp and AMP from ATP and GTP. Subsequently, AMP-Glo^™^ Reagent I was added to each reaction well and incubated for 1 h at room temperature to terminate the enzymatic reaction, simultaneously remove ATP, and convert AMP to ADP. The AMP-Glo™ Reagent II, along with Kinase-Glo One solution, was used to prepare the AMP detection solution as instructed in the assay protocol upon completion of the first step of incubation and was added to the reaction wells immediately after preparation. The reactions were incubated at room temperature for 1 h, and the luminescence intensity was measured using a BioTek Synergy plate reader (Agilent, Santa Clara, CA, USA).

### 4.6. Hydrolysis Assay

The hydrolysis of (p)ppGpp results in the formation of GTP and PPi. PhosphoWroks™ Pyrophosphate Assay Kit (AAT Bioquest, Pleasanton, CA, USA) provides the spectrophotometric method for measuring pyrophosphate. The kit uses proprietary fluorogenic pyrophosphate sensor that emits fluorescence intensity, proportional to the concentration of pyrophosphate. All the working solutions were prepared as per the instructions in the manufacturer’s protocol. Subsequent reactions detailed below for this assay were all carried out on a Corning 96-well flat-bottom black plate (Corning, NY, USA). Standard curve was generated with pyrophosphate in the concentration range from 100 µM to 0.014 µM and serially diluted in 50 mM Tris at pH 8.5, 350 mM NaCl, and 5% glycerol. The recombinant proteins were tested at a concentration of 0.5 µM in the reaction buffer containing 100 µM of (p)ppGpp and 100 µM of MgCl_2._ The hydrolysis reaction mixture was incubated at room temperature for 40 min. Thereafter, 50 µL of PPi Sensor working solution was added to all the wells and incubated at room temperature for 30 min. The fluorescence intensity was monitored at E_x_/E_m_ = 370/470 nm.

### 4.7. Tryptophan Fluorescence Quenching Spectroscopy

Steady-state fluorescence measurements were performed with the Cary Varian Eclipse fluorescence spectrophotometer (Leine, Germany), using a 10 mm path-length quartz cuvette. Both excitation and emission slit widths were set to 5 nm. The binding affinity of tRNA to *Mav*Rel TGS was determined by tryptophan fluorescence quenching titration. Purified *Mav*Rel TGS (60 μM) and *Mav*Rel Y473W (3 μM) were titrated in 50 mM Tris-HCl, pH 8.5, 350 mM NaCl, and 1 mM DTT, with increasing concentrations of tRNA, while quenching of tryptophan fluorescence was monitored at 355 nm following excitation at 295 nm. Dissociation constant (*K_d_*) and maximum fluorescence (ΔF_max_) values were determined following fitting of the data to an equation describing binding to a single affinity site.

### 4.8. NMR Spectroscopy Data Acquisition and Backbone Assignment

All NMR data acquisition were performed on a Bruker Avance 700 MHz spectrometer (Karlsruhe, Germany) equipped with cryoprobe at 298 K, using ^15^N and ^13^C labeled *Mav*Rel ACT, with concentration of 0.3 mM in buffer consisting of 50 mM Tris, pH 8.5, 350 mM NaCl, 5% glycerol, 1 mM DTT, and 10% D_2_O. A standard 2D ^1^H-^15^N HSQC spectrum and 3D heteronuclear NMR data were recorded. Three-dimensional triple resonance spectra of HNCA, HNCACB, CBCA(CO)NH, and HN(CO)CA were collected in nonuniform sampling (NUS [68]) of the indirect dimension as 20% sampling rates. ^15^N ^1^HN, ^13^C_α_, and ^13^C_β_ assignments for the backbone of the *Mav*Rel ACT were carried out as described in [69]. NMR spectra were processed using Topspin (Bruker BioSpin, Ettlingen, Germany) and analyzed with SPARKY [70].

### 4.9. NMR Titration of MavRel ACT with Valine

Uniformly ^15^N labeled NMR sample of *Mav*Rel ACT (0.5 mM) was prepared in buffer containing 50 mM Tris, pH 8.5, 100 mM NaCl, 5% glycerol, and 10% D_2_O. A 400 mM valine stock solution was prepared in the same buffer. ^1^H-^15^N HSQC spectra for apo *Mav*Rel ACT and *Mav*Rel ACT with valine at the molar ratio of 1 (dimer):10 and 1 (dimer):50 were recorded on a Bruker 700 MHz NMR spectrometer. The pH was not adjusted after the addition of the valine as the stock prepared for the amino acid was prepared in the same buffer as the protein. Chemical shift perturbations were studied to detect binding between *Mav*Rel ACT and valine.

## Data Availability

The original contributions presented in this study are included in the article and Supplementary Materials. Further inquiries can be directed to the corresponding author.

## Supplementary Materials

The following supporting information can be downloaded at: https://www.mdpi.com/article/10.3390/antibiotics14100964/s1. Figure S1: Illustration of *Mav*Rel domains studied and their respective residue numberings. Figure S2: SDS-PAGE validation and valine binding assay of *Mav*Rel Y473W via tryptophan quenching fluorescence spectrometry. Table S1: Primer sequences and their respective template used for the amplification of the corresponding plasmids.
