# Supplementary material for: The N-Terminal Extension of the Mycobacterium avium Rel Protein Is a Dual Regulator of the Bifunctional Enzyme and Represents a Novel Target
Source: Antibiotics (Basel). 2025 Sep 25;14(10):964. doi: 10.3390/antibiotics14100964 (PMC12561398; doi:10.3390/antibiotics14100964)
Supplement: Supplementary file 1 [file antibiotics-14-00964-s001.zip › antibiotics-3813865-supplementary.pdf]

antibiotics

MDPI

Supplementary Information

Supplementary Figure S1:

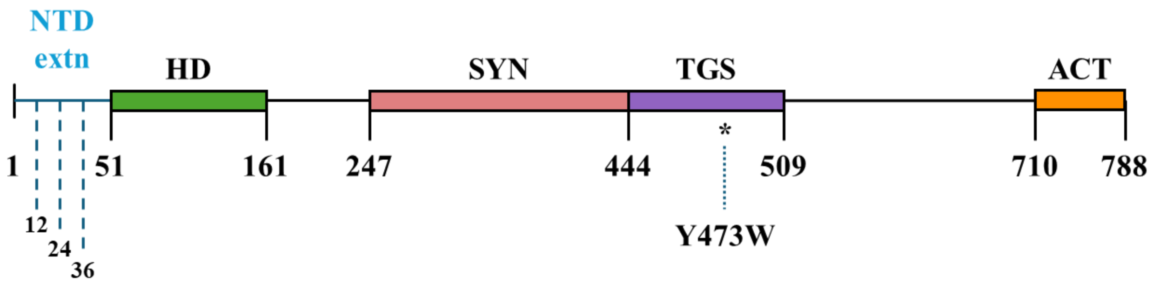

Illustration of all *Mav*Rel domains assayed in this study and the residue numbering of each domain.

Supplementary Figure S2:

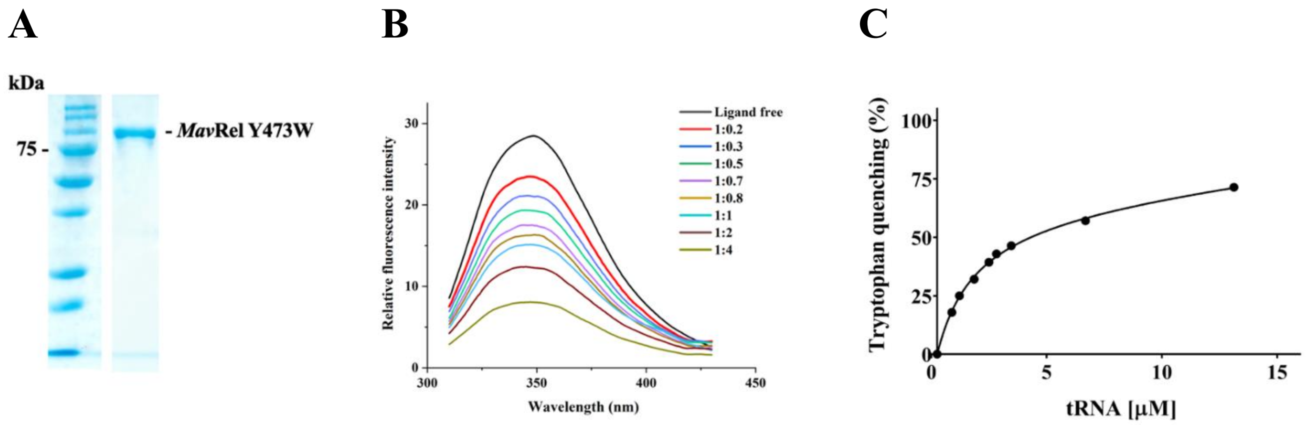

A) SDS-PAGE gel of purified *Mav*Rel Y473W. **(B)** Tryptophan fluorescence intensity measured for *Mav*Rel Y473W in the absence and presence of tRNA, titrated at increasing concentrations. **(C)** Tryptophan quenching measurement at increasing concentrations of tRNA (0-12 µM) for tRNA binding constant determination.

Supplementary Table S1:

Table shows the primer sequences and its respective template used for the amplification of the corresponding plasmids.

	**Template**	**Primer 1**	**Primer 2**
*Mav*Rel$_{1\text{-}161}$	pET29b-*Mav*Rel$_{1\text{-}788}$	ACAAAACTTTAGCTCGAGCACCACCACCACCACCACTG	GCTCGAGCTAAAGTTTTGTGACACCATCAACAAGGTGTCCAACT
*Mav*Rel$_{13\text{-}444}$	pET29b-*Mav*Rel$_{1\text{-}444}$	CCACCACGCACCAGCAGAATCTCCCCCG	GCTGGTGCGTGGTGGTGGTGGTGGTGCATC
*Mav*Rel$_{25\text{-}444}$	pET29b-*Mav*Rel$_{1\text{-}444}$	CACCACCACACACCGGAACCGCCAACA	TCCGGTGTGTGGTGGTGGTGGTGGTGCATC
*Mav*Rel$_{37\text{-}444}$	pET29b-*Mav*Rel$_{1\text{-}444}$	CACCACCACTCCAGTGCCAGTCGGCGTG	GGCACTGGAGTGGTGGTGGTGGTGGTGCATC
*Mav*Rel$_{51\text{-}444}$	pET9d-*Mav*Rel$_{51\text{-}788}$	GCGCTATGACGGATCCGGCTGCTAACAAAGC	GGATCCGTCATAGCGCAGCGACTCGAGGA
*Mav*Rel$_{51\text{-}509}$	pET9d-*Mav*Rel$_{51\text{-}788}$	TTTCACCGGATCCGGCTGCTAACAAAGCCCG	GGATCCGGTGAAAACCTCGACGACTTCCCCGTTTTCC
*Mav*Rel$_{445\text{-}509}$ (TGS)	pET29b-*Mav*Rel$_{1\text{-}788}$	CACCACCACCTGGCAGTCCAGGAAATATTTGTATTTACACCA	CTGCCAGGTGGTGGTGGTGGTGGTGCATCATA
		TTTACTTGAGATCCGGCTGCTAACAAAGCCC	CCGGATCTCAAGTAAAGACTTCAACTACCTCTCCATTCTC
*Mav*Rel TGS$_{Y473W}$	pET29b-*Mav*Rel$_{1\text{-}788}$	GATTTTGCGtggGCAGTCCATACTGAAGTTGGCCAT	GACTGCccaCGCAAAATCAATAGGTGTACTGCCCGTC
*Mav*Rel$_{710\text{-}788}$ (ACT)	pET29b-*Mav*Rel$_{1\text{-}788}$	CACCACCCTTCCCCTAGTTCCGTTTTCCTTGTTGCGA	AGGGGAAGGGTGGTGGTGGTGGTGGTGCATCATAT
*Mav*Rel Y473W	pET29b-*Mav*Rel$_{1\text{-}788}$	GATTTTGCGtggGCAGTCCATACTGAAGTTGGCCAT	GACTGCccaCGCAAAATCAATAGGTGTACTGCCCGTC
